# Identification of a Classical Mutant in the Industrial Host *Aspergillus niger* by Systems Genetics: LaeA Is Required for Citric Acid Production and Regulates the Formation of Some Secondary Metabolites

**DOI:** 10.1534/g3.115.024067

**Published:** 2015-11-13

**Authors:** Jing Niu, Mark Arentshorst, P. Deepa S. Nair, Ziyu Dai, Scott E. Baker, Jens C. Frisvad, Kristian F. Nielsen, Peter J. Punt, Arthur F.J. Ram

**Affiliations:** * Molecular Microbiology and Biotechnology, Institute of Biology Leiden, Leiden University, 2333 BE, Leiden, The Netherlands; † Chemical and Biological Process Development Group, Pacific Northwest National Laboratory, Richland, Washington 99352; ‡ Environmental Molecular Sciences Laboratory, Pacific Northwest National Laboratory, Richland, Washington 99352; § Department of Systems Biology, Technical University of Denmark, 2800 Kgs Lyngby, Denmark; ** Dutch DNA, 3700 AJ Zeist, The Netherlands

**Keywords:** organic acids, filamentous fungi, bulk segregant analysis, parasexual cycle, genome sequencing

## Abstract

The asexual filamentous fungus *Aspergillus niger* is an important industrial cell factory for citric acid production. In this study, we genetically characterized a UV-generated *A. niger* mutant that was originally isolated as a nonacidifying mutant, which is a desirable trait for industrial enzyme production. Physiological analysis showed that this mutant did not secrete large amounts of citric acid and oxalic acid, thus explaining the nonacidifying phenotype. As traditional complementation approaches to characterize the mutant genotype were unsuccessful, we used bulk segregant analysis in combination with high-throughput genome sequencing to identify the mutation responsible for the nonacidifying phenotype. Since *A. niger* has no sexual cycle, parasexual genetics was used to generate haploid segregants derived from diploids by loss of whole chromosomes. We found that the nonacidifying phenotype was caused by a point mutation in the *laeA* gene. *LaeA* encodes a putative methyltransferase-domain protein, which we show here to be required for citric acid production in an *A. niger* lab strain (N402) and in other citric acid production strains. The unexpected link between LaeA and citric acid production could provide new insights into the transcriptional control mechanisms related to citric acid production in *A. niger*. Interestingly, the secondary metabolite profile of a Δ*laeA* strain differed from the wild-type strain, showing both decreased and increased metabolite levels, indicating that LaeA is also involved in regulating the production of secondary metabolites. Finally, we show that our systems genetics approach is a powerful tool to identify trait mutations.

*Aspergillus niger* is a biotechnologically important filamentous fungus and is used as an industrial cell factory for the production of organic acids and enzymes (Pel *et al.* 2007; Andersen *et al.* 2011). A key characteristic of *A. niger* is the rapid acidification of the culture medium during exponential growth owing to the secretion of mainly gluconic acid, citric acid, and oxalic acid, resulting in a pH below 2.0 in uncontrolled batch cultures. Medium acidification has some important consequences for the behavior of *A. niger* as a cell factory because both organic acid production and enzyme production are highly dependent on the ambient pH. For further reading about the metabolic pathways involved in organic acid biosynthesis we refer to two recent reviews (Kubicek and Karaffa 2010; Li and Punt 2013). The genome sequence of the citric acid production wild-type strain (ATCC1015) has been determined, and a spontaneous mutant of this strain (ATCC11414) was used for subsequent studies of citric acid production (Perlman *et al.* 1946; Baker 2006; Andersen *et al.* 2011). Organic acid production is highly dependent on medium composition and, interestingly, also on the environmental pH. Under laboratory conditions using bioreactor-controlled fermentation, the pH can be maintained at a fixed value, and this has revealed that the production of specific organic acids is clearly pH-dependent. Citric acid production is optimal at low pH (2.0) (Karaffa and Kubicek 2003; Magnuson and Lasure 2004), and requires high glucose and low manganese concentrations (de Ruijter *et al.* 1999; Andersen *et al.* 2009). Oxalic acid production is most efficient between pH 5.0 and 8.0, and absent at pH 3.0 (de Ruijter *et al.* 1999; Andersen *et al.* 2009). Production of gluconic acid is also pH-dependent and optimal at pH 6.0, but absent at pH 2.5 (Andersen *et al.* 2009). Gluconic acid and citric acid can be metabolized by *A. niger*, while oxalic acid is not taken up and metabolized and accumulates in the medium (Poulsen *et al.* 2012). Therefore, oxalic acid accumulation is the main cause of acidification of the medium during the late growth phases of batch cultures. Indeed, an *A. niger* mutant in which oxalic acid synthesis was abolished through inactivation of the oxaloacetate hydrolase (*oahA*) gene behaves as a nonacidifying mutant (Pedersen *et al.* 2000; Andersen *et al.* 2009; Li *et al.* 2013).

Ambient pH is also an important environmental factor influencing the expression of extracellular enzymes (van den Hombergh *et al.* 1996; Peñalva and Arst 2002). As a saprophytic fungus, *A. niger* is well known for its ability to secrete enzymes that are required for the decay of organic plant-derived polysaccharides and proteins. The influence on ambient pH on protease production has been studied in more detail and it has been shown that at pH 4.0 and lower, protease activity is high. Protease activities are lower at pH 5.0 and decrease further at pH 6.0 (Braaksma and Punt 2008; Braaksma *et al.* 2009). The genes encoding the major extracellular proteases *pepA* and *pepB* are induced under acidic conditions (Jarai and Buxton 1994; van den Hombergh *et al.* 1997a, 1997b). The regulation of proteases is not only dependent on ambient pH but is also controlled by nutrient conditions, including nitrogen source and carbon availability (Braaksma *et al.* 2009; van den Hombergh *et al.* 1997a). Several wide-domain transcription factors are involved, including the nitrogen regulator AreA (MacCabe *et al.* 1998; Lenouvel *et al.* 2001), the general repressor TupA (Schachtschabel *et al.* 2013), the carbon repressor protein CreA, and PacC (Fraissinet-Tache *et al.* 1996). In *A. nidulans*, PacC is a transcriptional activator of alkaline-induced genes and a repressor of acid-induced genes (Peñalva and Arst 2002). Analysis of the expression of protease genes in *pacC* mutants of *A. niger* has indicated the involvement of PacC in the regulation of *pepA* and *pepB* (Fraissinet-Tache *et al.* 1996). In addition, a gene encoding a protease-specific, positive-acting transcription factor required for the induction of several protease-encoding genes, including *pepA* and *pepB*, has been identified. A strain carrying a mutation in this gene, *prtT*, was identified in a mutant screen for protease-minus mutants (Punt *et al.* 2008). The *prtT* gene encodes a Zn(II)_2_Cys_6_ transcription factor and controls (in combination with the wide-domain regulators CreA, AreA, TupA, and PacC) the expression of protease genes.

The *prtT* mutant was isolated by a classical forward genetic mutant screen for protease mutants (Mattern *et al.* 1992). Such screens are still powerful tools to identify new and unexpected gene functions. To identify the gene mutated in a particular mutant, complementation analysis with genomic libraries is traditionally used. Such a genomic cosmid library is also available for *A. niger* and has been successfully used before (*e.g.*, Punt *et al.* 2008; Damveld *et al.* 2008; Meyer *et al.* 2009). However, several problems are encountered in identifying complementation mutants, either because the gene might be lacking in the library or because of complications in screening thousands of transformants for complementation. Whole genome sequencing is an alternative method for identifying the mutation that is responsible for a particular phenotype. As classical mutagenesis might also result in mutations unrelated to the phenotype, several researchers have used bulk segregant analysis to identify the relevant mutations. This method was first developed in plant genetics (Michelmore *et al.* 1991) and, subsequently, used in combination with next-generation sequencing in various other organisms including *Saccharomyces cerevisiae* (Wenger *et al.* 2010; Dunham 2012), filamentous fungi (Pomraning *et al.* 2011; Nowrousian *et al.* 2012; Bok *et al.* 2014), and insects (Park *et al.* 2014). In this approach, the mutant of interest is crossed to a wild-type strain, haploid segregants displaying the phenotype of interest are pooled, and DNA from this pool of segregants is sequenced using deep sequencing (*e.g.*, Illumina). In addition to the pooled segregants, the two parental strains from the cross are sequenced, and single-nucleotide polymorphism (SNP) analysis between the two parental strains is performed. The SNP that causes the phenotype will be conserved in all the progeny displaying the phenotype (homozygous SNP), while mutations that are not related to the phenotype will have a 50% chance to be present in the genomic DNA of the pool (heterozygous SNPs). SNPs that are located close to the mutations of interest will cosegregate and can only separate via recombination. Since *A. niger* lacks a sexual cycle, we have used the parasexual cycle of *A. niger* (Pontecorvo *et al.* 1953; Bos *et al.* 1988) to generate a pool of segregants. In this study, we have used bulk segregant analysis combined with Illumina sequencing to characterize the nonacidifying D15 mutant of *A. niger*. Here, we show that a mutation in the *laeA* gene causes the nonacidifying phenotype of the D15 mutant, and that the loss of *laeA* strongly affects the production of secondary metabolites in *A. niger*.

## Materials and Methods

### Strains, media, and molecular methods

**Figure 1:**
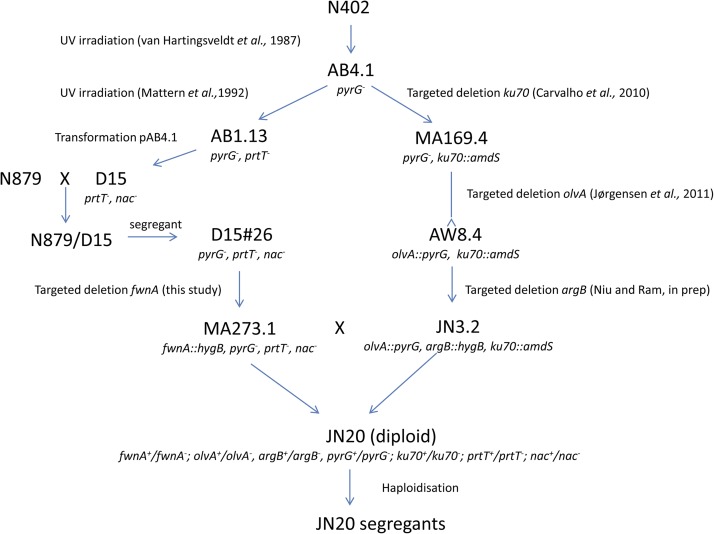
Schematic overview of the lineage of the D15 mutant and its derivatives. The genotypes of the strains are given in Table 1.

*A. niger* strains used in this study are listed in Table 1. Because of the complexity of the strain background of the D15 mutant, a schematic overview of the strain lineages is given in Figure 1. Strains were grown on minimal medium (MM) (Bennett and Lasure 1991) containing 1% (w/v) glucose, or on complete medium (CM) containing 2% (w/v) glucose, 0.5% (w/v) yeast extract, and 0.1% (w/v) casamino acids in addition to MM. When required, plates or medium were supplemented with 10 mM uridine or 0.2 mg/ml arginine. Plates were incubated at 30°. Skimmed milk, MacConkey agar plates to assay acidification contained MM + glucose medium without nitrate (ASP-N) (Arentshorst *et al.* 2012) supplemented with 1% skimmed milk (Difco) and 2% MacConkey agar. Preacidified (pH 3.0) skimmed milk, MacConkey agar plates were used to assay protease activity. The pH was set at 3.0 by the addition of hydrogen chloride. Citric acid production (CAP) medium was prepared as described previously (Dai *et al.* 2004).

**Table 1:** *Aspergillus niger* strains used in this study

Strain	Description	Reference
N402	*cspA1* derivative of ATCC9029	Bos *et al.* 1988
N879	*fwnA1*, *argH12*, *pyrA5*, *leuA1,pheA1*, *lysD25*, *oliC2,crnB12*	Bos *et al.* 1993
AB4.1	*pyrG378* in N402	van Hartingsveldt *et al.* 1987
AB1.13	*prtT-13*, *pyrG378*	Punt *et al.* 2008
AB1.13-*pyrG^+^*	*prtT-13*	Punt, unpublished
MA169.4	Δ*kusA*::*DR_amdS_DR*, *pyrG378*	Carvalho *et al.* 2010
D15#26	*prtT-13*,, *pyrG378*, nonacidifying	This study
D15#26-*pyrG^+^*	*prtT-13*, nonacidifying	Punt, unpublished
MA273.1	*prtT-13*, *pyrG378*, Δ*fwnA*::*hygB*, nonacidifying	This study
JN26.1	*prtT-13*, *pyrG378*, Δ*fwnA*::*hygB* nonacidifying, pAO4-13-LaeA	This study
AB1.13∆oahA#76	Δ*oahA*::*pyrG#76* in AB1.13	Li *et al.* 2013
AW8.4	*olvA*::*pyrG* in MA169.4	Jørgensen *et al.* 2011
JN3.2	*argB*::*hygB* in AW8.4	Jing, unpublished
JN20	Diploid MA273.1 x JN3.2	This study
JN21.1	D15#26 pAO4-13	This study
JN22.7	D15#26 pAO4-13-LaeA	This study
JN24.6	Δ*laeA* in AB4.1*kusA*::*AfpyrG*	This study
KB1001	*kusA*::*pyrG*	Chiang *et al.* 2011
KB1001Δ*laeA*	Δ*laeA*::*hygB* in KB1001	This study

Amplification of plasmid DNA was performed using the XL1-Blue strain, which was transformed using the heat-shock protocol as described (Inoue *et al.* 1990). Transformation of *A. niger* was performed as described by Arentshorst *et al.* (2012), using 40 mg lysing enzyme (L-1412, Sigma-Aldrich, St. Louis) per g wet weight of mycelium. *A. niger* genomic DNA was isolated as described previously (Arentshorst *et al.* 2012). The (α-^32^P)dCTP-labeled probes were synthesized using the Rediprime II DNA labeling system (Amersham Pharmacia Biotech, Piscataway, NJ), according to the instructions of the manufacturer. All molecular techniques were carried out as described previously (Sambrook *et al.* 1989). Sequencing was performed by Macrogen Europe (Amsterdam, The Netherlands).

### Construction of plasmids and strains

D15#26 was transformed with the *fwnA*::*hygB* disruption plasmid (Jørgensen *et al.* 2011) to generate MA273.1 (*prtT-13*, *pyrG378*, Δ*fwnA*::*hygB*, nonacidifying). Strain JN3.2 (*olvA*::*pyrG*, *argB*::*hygB*) was obtained by disrupting the *argB* gene of *A. niger* (Lenouvel *et al.* 2001) in AW8.1 (Jørgensen *et al.* 2011). Details for the disruption of *argB* in JN6.2 will be published elsewhere (J. Niu and A. F. J. Ram, unpublished results).

Disruption of the *laeA* gene (An01g12690) in the N402 background was carried out using the split-marker approach (Arentshorst *et al.* 2015). The 910 bp-long 5′-flank and 901 bp-long 3′-flank regions were amplified using the primers listed in Supporting Information, Table S1. These PCR fragments were used in a fusion PCR with the *A. oryzae pyrG* gene (pAO4-13) (de Ruiter-Jacobs *et al.* 1989) to generate the split-marker fragments. After amplification, the 5′­flank-*pyrG* and 3′­flank-*pyrG* fragments were purified from the agarose gel and simultaneously transformed to the recipient *A. niger* strain AB4.1. Putative *laeA* disruption strains were purified by two consecutive single colony streaks. Genomic DNA was isolated as described (Arentshorst *et al.* 2012) and Southern blot analysis was performed to confirm proper deletion. JN24.6 was used for further experiments.

The Δ*laeA* mutant strain in the ATCC11414 background was generated by homologous replacement of *laeA* in the *ATCC*11414 Δ*kusA* derivative (Chiang *et al.* 2011). The *laeA* deletion cassette was constructed by PCR amplification of upstream and downstream regions of the *A. niger laeA* gene using primers listed in Table S2. The hygromycin resistance marker was amplified from pCB1003 (Fungal Genetics Stock Center) by PCR using the oligonucleotides hph5 and hph3 (Table S2). The DNA fragments were assembled into the backbone plasmid vector of pBlueScript II SK(-), linearized with restriction endonucleases *Hind*III and *Pst*I using the Gibson assembly cloning kit (New England Biolabs). The assembled plasmid DNA was transferred into Top10 *Escherichia coli* competent cells by lithium acetate-mediated transformation (Life Technologies). The transformed bacterial colonies were screened for DNA fragment insertion by restriction endonuclease digestion with *Pvu*II and *Xho*I. The Δ*laeA* cassette was isolated from plasmid DNA by digestion with endonucleases *Hind*III and *Xba*I for *A. niger* transformation. After purification of hygromycin-resistant transformants, proper *laeA* deletion strains were identified via diagnostic PCR using primers laeAsc5 and laeAsc3 (Table S2).

The vector for complementing the nonacidifying phenotype of the D15 mutant (pJN33) was made by amplifying the *laeA* gene, including promoter and terminator sequences, with primers *laeA*(*EcoR*I)5f and *laeA*(*EcoR*I)6r. The 3139 bp-long PCR fragment was then cloned into pJet1.2 (blunt-end cloning vector) and this was verified by DNA sequencing. Subsequently, the PCR fragment was excised from pJet1.2*-laeA* using *EcoR*I and inserted into *EcoR*I-digested plasmid pAO4-13 to give pJN33 and transformed to the recipient *A. niger* strains MA273.1 and D15#26. JN24.6 was complemented using the same vector by performing cotransformation with the hygromycin resistance gene-containing plasmid pAN7.1.

To sequence the *oahA* gene in the D15 mutants, two primers (Table S1) were designed to amplify the open reading frame including 1 kb flanking regions. The PCR fragment was cloned in pJet2.1 and fully sequenced.

### A. niger genetics and analysis of segregants

Parasexual crossings were performed as described (Bos *et al.* 1988), with minor modifications. Selecting of a balanced heterokaryon of a cross between MA273.1 (*prtT-13*, *pyrG378*, Δ*fwnA*::*hygB*, nonacidifying) and JN3.2 (*olvA*::*pyrG*, *argB*::*nicB*) was performed on MM after pregrowth of both strains for 36 hr in 0.5 ml CM containing uridine and arginine. The mycelial mat was fragmented using toothpicks and incubated for 7 d on MM. Spores from heterokaryotic mycelium were carefully isolated to prevent fragmentation of the mycelia, filtered over a double miracloth filter and plated out on selective MM. Using two color marker-containing, haploid strains, we could identify diploids visually by selecting colonies that exclusively formed black spores. A resulting diploid (JN20) was haploidized by adding benomyl (0.6 µg/ml) to CM supplemented with uridine and arginine. Haploid segregants (fawn- or olive-colored sectors) were purified and genotypically analyzed for conidial spore color, *pyrG* and *argB* auxotrophies, acidification, and protease production. Nonacidifying segregants were collected and, in total, 140 nonacidifying segregants were obtained. Seventy-eight segregants were individually grown in complete medium and, from each strain, 200 mg fresh weight mycelia were collected for genomic DNA isolation. Mycelia of ∼20 strains (4 g of mycelia) was mixed and ground, and genomic DNA was isolated. Equal amounts of DNA of each of the four pools was pooled together to obtain the genomic DNA pool for sequencing. Genomic DNA from D15#26 and JN3.2, and the pools was further purified using Macherey-Nagel NucleoBond Xtra columns and used for DNA sequencing.

### DNA sequencing and data analysis

Illumina paired-end sequencing was performed by ServiceXS using Illumina kits (cat# 1001809 and 1005063) and protocols according to the instructions provided by the supplier. The quality and yield after sample preparation were checked and were consistent with the expected size of 300 bp after excision from the gel. Clustering and DNA sequencing using Illumina cBot and HiSequation 2000 were performed according to manufacturer’s protocols. Two sequencing reads of 100 cycles each using Read1 and Read2 sequencing primers were performed with the flow cell. For strains MA273.1 and JN3.2, 4.0 Gb of DNA sequence were obtained. Two separate pools of segregant DNA, consisting of 10.3 and 13.4 Gb of DNA sequence, respectively, were separately sequenced. All raw high-throughput sequence data will be deposited in the SRA database. Image analysis, base calling, and quality check were performed with the Illumina data analysis pipeline RTA v1.13.48 and/or OLB v1.9/CASAVA v1.8.2. Based on the mapped reads, variants in the sample data were detected by comparison with the reference genome of ATCC1015 (http://genome.jgi-psf.org/pages/search-for-genes.jsf?organism=Aspni5), and between the samples by using an in-house SNP pipeline v3.2 (ServiceXS). Validated variants must be consistently found in one location in at least one sample with a frequency of 0.7 or higher, in at least 20 overlapping reads (minimum coverage) with no quality filtering, before it is reported as a SNP. The combined pool sample (23.7 Gb) was processed with a minimal variant frequency of 0.3. For each SNP, it was verified whether the SNP was in a predicted protein-encoding region using the *A. niger* 3.0 genome at JGI and the SNP coordinates.

### Culture conditions and metabolite analysis

Controlled bioreactor cultivations for *A. niger* N402 and D15#26 were performed as previously described, using fixed pH values varying from pH 2 to pH 7 (Braaksma *et al.* 2009). Organic acid analyses were performed as described previously (Li *et al.* 2013). Shake flask cultures containing 50 ml of MM were inoculated with 5 × 10^7^ spores and incubated at 30° at 150 rpm. For each sampling time point, an individual flask was inoculated to determine biomass accumulation, and culture pH, and to sample medium for acid and metabolite analysis. Protease activities of culture medium samples were measured using the P-check assay at pH 2.7 according to the supplier’s instructions (Jena BioScience). Broth samples of N402, AB1.13, D15 and Δ*laeA* taken at 96 hr were analyzed for secondary metabolite production. A 5.0 ml sample of fermentation broth (including biomass) was diluted with 5.0 ml isopropanol (LC-MS grade, Sigma-Aldrich), placed in an ultrasonication bath for 20 min, and centrifuged at 4000 × g for 5 min. A 1ml subsample was transferred to a 2 ml HPLC vial. For secondary metabolite analysis, N402, AB1.13, D15 and Δ*laeA* were grown on YES or CYA agar in darkness at 25° for 7 d, 3 plugs of approx. 0.6 cm^2^ culture were sampled and extracted using ethyl-acetate-dichloromethane-methanol, evaporated to dryness, and redissolved in methanol (Nielsen *et al.* 2009).

Samples were then analyzed by liquid chromatography-high resolution mass spectrometry on Agilent 1290 infinity UHPLC (Agilent Technologies, Torrence, CA) equipped with an Agilent Poroshell 120 phenyl-hexyl column (250 mm × 2.1 mm, 2.7 µm particles), running an acidic water/acetonitrile gradient. This was coupled to an Agilent 6550 Q-TOF-MS equipped with an ESI source and operated in positive polarity, and sampling m/z 50-1700 in full scan and auto MS/MS mode (Kildgaard *et al.* 2014). Compounds were then identified by MS/HRMS spectra and retention time (Kildgaard *et al.* 2014), and peaks integrated using Agilent Quant Analysis 6.0 as described (Nielsen and Larsen 2015).

Cultivation to measure citric acid production under citric acid production conditions were performed in glass baffled shake flasks of 250 ml, which were silanized with 200 ml of a 5% solution of dichlorodimethylsilane in heptane to minimize leaching of metals. *A. niger* strains were grown in 75 ml CAP media containing 10 ppb Mn^2+^ at 30° and 200 rpm. Samples for citric acid analysis were taken after 5 d of growth. Citric acid concentrations were determined with an end-point spectrophotometric enzyme assay as described previously (Bergmeyer 1985) using 5 µl of each culture supernatant.

### Data availability

Strains and DNA sequence data are available upon request.

## Results

### Isolation of a nonacidifying A. niger strain D15#26

**Figure 2:**
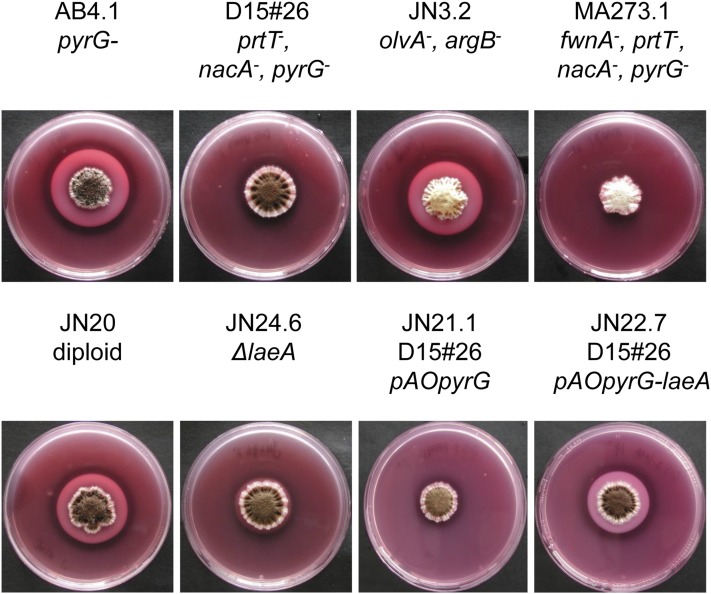
MacConkey agar milk plates for assaying medium acidification. The milk powder in MacConkey agar milk plates remains soluble at pH > 5.0. Acidification around the colony results in precipitation of the skimmed milk.

In a gene-expression study aimed at overproduction of bacterial levansucrase using cotransformation of low-protease *A. niger* mutant AB1.13 (Mattern *et al.* 1992; Punt *et al.* 2008) with the *A. niger pyrG* gene, a nonacidifying *A. niger* transformant showing increased growth on medium with inulin as a sole carbon source was isolated (E. Wanker and P. Punt, unpublished results). Acidification of the medium by *A. niger* can be easily visualized using MacConkey agar milk plates. These plates contain dissolved milk powder; they are clear at the initial pH of about 5, but form a white precipitate when the pH in the plate decreases to below 4.0. Growth of the wild-type strain and accompanying acidification of the medium results in a white precipitate around the colony while no precipitate is formed in the D15 mutant (Figure 2).

The mutant, displaying a nonacidifying phenotype, was crossed to *A. niger* strain N879 and a nonacidifying *pyrG*^-^, *prtT*^-^ segregant (D15#26) was selected for further studies. Southern analysis of this segregant showed that this segregant did not carry any additional remnants of the *pyrG* gene copies used in the transformation experiment that gave rise to strain D15 (data not shown). Another effect of the reduced acidification of this strain was that the total protease activity was further reduced compared to the low-protease host strain AB1.13. Culture pH and total proteolytic activities of batch-cultured N402, AB1.13 and D15 strains were analyzed in time. As shown in Table 2, the pH of the culture medium of the D15 strain remained around 6.5, whereas the N402 and the AB1.13 strains showed typical acidification of the medium. Proteolytic activity in the culture medium was assayed using the P-check assay. Proteolytic activity was reduced in the AB1.13 mutant and further reduced to about 10% of the wild-type level in the D15 mutant (Table 2).

**Table 2:** Culture pH and relative protease activity during batch growth

		24 hr		48 hr		72 hr		96 hr		120 hr	
Strain	Phenotype	pH	Relative Protease Activity*^a^*	pH	Relative Protease Activity	pH	Relative Protease Activity	pH	Relative Protease Activity	pH	Relative Protease Activity
N402	—	3.7	39%	4.4	57%	4.6	89%	5.2	79%	5.3	100%
AB1.13*^a^*	*prtT^−^*	3.5	23%	4.1	25%	4.0	38%	4.3	38%	4.8	48%
D15#26*^a^*	*prtT^−^*, *nac^−^*	6.3	5%	6.6	5%	6.3	9%	6.6	16%	6.8	10%

a Relative protease activity expressed as percentage of the protease activity in the culture fluid of wild-type (N402) after 120 hr of growth. Protease acidity was determined using the P-check assay. For the growth experiments, *pyrG*^+^ (uridine-prototrophic strains) were cultivated. *nac^-^* = nonacidifying.

### The nonacidifying phenotype in D15 is not caused by a mutation in the oahA gene

A low-protease, nonacidifying *A. niger* mutant was previously isolated by van den Hombergh and coworkers (van den Hombergh *et al.* 1995). This mutant, named *prtF*, lacks oxaloacetate acetylhydrolase activity, and it was shown that this strain was mutated in the *oahA* gene (Ruijter *et al.* 1999). Linkage analysis assigned the *prtF* mutation to linkage group V (van den Hombergh *et al.* 1995). Linkage analysis of the D15 mutant, by carrying out a parasexual cross with tester strain N879 (Table 1), revealed that the nonacidifying phenotype was linked to the *argH*12 marker on linkage group II (12.5% recombination) (P. J. Punt, unpublished results), indicating that the two mutants are affected in different loci. To make sure that the *oahA* gene was not mutated in the D15 mutant, the *oahA* gene (An10g00820), including 1000 nucleotide-flanking regions, was PCR-amplified from D15 and sequenced. No mutation in the gene was found, indicating that the mutation in D15 is not located in the *oahA* locus.

### Physiological analysis of the D15 mutant

The nonacidifying phenotype of the D15 mutant was compared with N402 and a Δ*oahA* mutant (Li *et al.* 2013) during batch growth using shake flask cultures. During growth, the unbuffered medium of the wild-type strain acidified quickly to reach a pH value of 3.5. At later time points (72 hr after inoculation), the pH of the wild-type strains stabilized around 5.4. The pH of the culture medium of the D15 mutant remained between 5.5 and 6.5 during the cultivation period (Table 2), while the pH of the *oahA* mutant strain increased from pH 4.5 to pH 8 (data not shown). HPLC analysis of the medium samples at different time points confirmed that the levels of citric acid and oxalic acid were reduced at the different time points in the D15 strain, whereas in the *oahA* strain citric acid was produced at even higher levels than in N402, and no oxalic acid was produced (data not shown). These physiological results also show that the genotype of D15 differs from the *oahA* mutant.

To analyze the profile of organic acids produced during controlled batch growth, the N402 strain and the D15 strain were cultivated in bioreactors at fixed pH values under the conditions described in the *Materials and Methods*. Since gluconic acid, oxalic acid, and citric acid are the main organic acids secreted into the medium, these acids were quantified by HPLC analysis. As shown in Table 3, the production of organic acids in the D15 mutant was strongly reduced. Production of citric acid was low in all samples and probably caused by high manganese concentrations and low glucose concentrations, both of which are known to diminish citrate production (Dai *et al.* 2004). Citric acid secretion was observed in N402 at all pH values, whereas no citric acid could be detected in the medium of the D15 mutant. At pH 3 and 4, oxalic acid was not detected in D15 medium, whereas gluconic acid levels were either similar (at pH 3.0) or reduced (at pH 4.0) compared to the wild-type (N402). At pH 5.0, 6.0, and 7.0, oxalic acid was again reduced in D15 medium compared to N402 medium. At these higher pH values, the D15 mutant produced similar amounts of gluconic acid. Growth of the N402 strain was severely reduced at pH 5.0 (2.8 g biomass/liter) in comparison to D15. At pH 5.0, the N402 strain produced high amounts of gluconic acid, and the base had to be added to maintain the pH at 5.0. At pH 5.0, the D15 mutant still grew relatively well (13 g biomass/liter) and did not secrete high amounts of gluconic acid. It should be noted that at pH 4.0 and 5.0, both strains also produced detectable levels of other unidentified acids. Based on these results, it is clear that the mutation in D15 caused considerable and complex physiological alterations in organic acid production, suggesting a mutation in a regulatory circuit governing primary metabolism. This hypothesis encouraged further research to elucidate the genetic background of the mutant strain.

**Table 3:** Physiological parameters of pH-controlled bioreactor cultivations of *A. niger* strains, and medium levels of the main three organic acids (gluconic, oxalic, and citric acid)

		pH Control		End of Glucose Consumption Phase			
	Cultivation	Use of	*EFT*	dwt	Gluconic	Oxalic	Citric
Strain	pH	Acid/Base	hr	g/L	Acid g/L	Acid g/L	Acid g/L
D15	3	Acid	45	15	1.3	n.d	n.d
N402	3	Acid	42	10.9	3.1	1.2	0.16
D15	4	Acid	49	17	1.0	n.d	n.d
N402	4	Acid	79	15	0.9	1.9	0.7
D15	5	Acid	42	13	12.6	0.5	n.d
N402	5	Base	49	2.8	30.5	4.3	1.6
D15	6	Base	42	2.4	36.4	0.8	n.d
N402	6	Base	48	1.8	32.2	4.4	0.16
D15	7	Base	48	2	39.0	1.6	n.d
N402	7	Base	63	1.7	43.2	3.4	1.2

EFT, elapsed fermentation time; dwt, dry weight; n.d, not detected.

### Isolation of segregants for bulk segregation analysis using next generation sequencing

To facilitate the isolation of a diploid strain to generate segregants for bulk sequencing analysis, mutant D15#26 was first transformed with the *fwnA*::*hygB* deletion cassette (Jørgensen *et al.* 2011). The *fwnA* gene encodes the polyketide synthase involved in conidial melanin synthesis, and a fawn-colored transformant was purified. This strain (MA273.1) produces fawn-colored conidiospores and also contains the *pyrG* auxotrophic marker. MA273.1 was crossed with JN3.2 (*olvA*::*pyrG*, *argB*::*hygB*). Using the complementary color markers (*fwnA* and *olvA*) and the complementary auxotrophies (*pyrG* and *argB*), a diploid was isolated from heterokaryotic mycelium. The resulting black-conidiating, prototrophic, diploid strain (JN20) acidified the medium, showing that the nonacidifying trait in D15 was recessive (Figure 2).

To obtain a collection of D15-derived segregants, diploid strain JN20 was point-inoculated on complete medium, supplemented with uridine and arginine, in the presence of benomyl. Benomyl affects microtubule dynamics, and growth of an *A. niger* diploid strain in the presence of sublethal concentrations of benomyl results in spontaneous haploidization by the loss of one of each pair of the eight chromosomes. The use of complementary spore color mutants allows easy identification of haploid sectors as these sectors display the spore color marker (Bos *et al.* 1988). From each point-inoculated diploid, a maximum of two segregants with different colors (fawn or olive) were purified. In total, 140 segregants were collected, purified, and analyzed for their spore color, *pyrG* and *argB* auxotrophies, acidification phenotype, and their protease production phenotype (Table S2). The possible genotypes of segregants and the number of segregants with the same genotype are presented in Table S3. First, we determined if all markers were more or less equally represented in the segregants. As shown in Table 4, roughly equal numbers of segregants were found for both alleles of the markers. The conidial color markers *fwnA* and *olvA* were localized on different arms of linkage group I, and no haploid recombinants producing black spores were isolated in our segregants. Two possible *fwnA/olvA* double mutants were detected in the segregants since such *fwnA* mutants are *pyrG^+^*, indicating that they might also harbor the *olvA*::*pyrG* disruption (Table S2). Table 5 presents the results from the marker linkage analysis. Because the *olvA* gene is disrupted by the *pyrG* gene, all *olvA* strains are *pyrG^+^*. The *argB* gene is on the same linkage group as the *olvA* marker (linkage group I), explaining the observed linkage of *olvA* and *argB*. We also noticed the strong coupling of the *fwnA*::*hygB* disruption with the *pyrG* gene. The *pyrG* is reported to be localized on the left arm of linkage group III, but our data show strong linkage between the *pyrG* marker and the *fwnA* marker (Table 5). Possibly, the *pyrG* gene in our strain is translocated to linkage group I, which could explain the linkage. Further research is required to clarify this, but the possible translocation has no effect on linkage analysis of the nonacidifying mutation in D15. The linkage analysis also showed that the nonacidifying phenotype is not linked to linkage group I (*fwnA*, *olvA*, and *argB*) and is not linked to the *prtT* mutation (linkage group VI) (Punt *et al.* 2008), as expected. From the 140 segregants, 78 displayed the nonacidifying phenotype, indicating that this phenotype is caused by a single mutation. These 78 segregants were individually grown and fresh weight mycelium of each strain was collected. Pooled mycelium of about 20 strains was used for genomic DNA purification. An equal amount of DNA from each of the four pools was combined to obtain the genomic DNA pool for sequencing.

**Table 4:** Distribution of marker alleles among the 140 segregants

Marker	# of Segregants		# of Segregants	
*fwnA^-^/olvA^-^**^a^*	64	*fwnA^-^*	76	*olvA^-^*
*pyrG*	78	*pyrG^+^*	62	*pyrG^-^*
*argB*	64	*argB^+^*	76	*argB^-^*
Nonacidifying	62	Acidifying	78	Nonacidifying
*prtT*	68	*prtT^+^*	72	*prtT^-^*

a Segregants are either *fwnA*^-^ or *olvA*^-^ due to the tight coupling of both markers even though the markers are located on two different sides of the centromere of chromosome III.

**Table 5:** Pairwise marker analysis of the diploid strain JN20 (MA273.1 (*fwnA^-^,* *pyrG^-^,* *argB^+^,* *nac^-^,* *prtT^-^*) x JN3.2 (*olvA^-^,* *pyrG^+^,* *argB^-^,* *nac^+^,* *prtT^+^*))

markers	*fwnA*	*olvA*	*pyrG^-^*	+	+	*argB^-^*	*nac^-^*	+	*prtT*	+
*fwnA*	64	0	1% 0%	1%			38%		47%	
*olv*	0	76								
										
*pyrG^-^*	62	0			2%		39%		48%	
+	2	76								
										
+	63	1	61	3			37%		47%	
*argB^-^*	1	75	1	75						
										
*nac^-^*	37	41	35	43	38	40			31%	
+	27	35	27	35	26	36				
										
*prtT*	26	46	24	48	26	46	47	25	PS	NPS
+	38	30	38	30	38	30	31	37	NPS	PS

The frequencies of pairwise gene combination are shown in the lower left half of the table. For each gene combination the number of parental segregants (PS) or nonparental segregants (NPS) are indicated in the top left/bottom right (PS) or top right/bottom left (NPS), respectively. In the upper right half, the recombination frequencies are given. Recombination frequencies are calculated as the number or nonparental segregants / total number of segregants × 100%; 140 segregants were analyzed.

### SNP analysis of parental strains and bulk segregants

The genomes of parental strains (MA273.1 and JN3.2) were sequenced and SNP analysis was performed as described in *Materials and Methods*. The reads were mapped to the genome sequence of the *A. niger* strain ATCC1015, as this strain is most similar to the N400/N402 background (Andersen *et al.* 2011). In total, 52 SNPs were identified between the two parental strains. We also expected to identify the mutation in the *prtT* gene, which was previously shown to be a single point mutation (T to C), causing an amino acid change [leucine (CTA) to proline (CCA)] in the PrtT protein (Punt *et al.* 2008). Indeed, as indicated in Table S4, we again found the SNP in the D15 mutant that is responsible for the *prtT* phenotype. Subsequently, we looked for homozygous SNPs within the pool of segregants. Theoretically, the mutation responsible for the phenotype should be completely conserved in the pools of segregants, whereas SNPs not related to the phenotype should have a 50% chance to be present. As shown in Table S4, three SNPs were found to be completely conserved. All three mapped to the right arm of linkage group II. Three other SNPs in linkage group II showed a high (∼98%), but not absolute conservation. Apparently, these SNPs are linked to our trait of interest, but a few segregants have been recombined between the SNP and our gene of interest and have, therefore, lost complete conservation. The linkage of the conserved SNPs to linkage group II is consistent with the observed linkage to linkage group II when crossed to marker strain N879 (see above). Further examination of the three SNPs that were fully conserved showed that only a single SNP at position 1762101 (G to C) was present in a protein-encoding region, corresponding to gene An01g12690. The protein encoded by this gene is the predicted ortholog of LaeA, a well-studied putative methyltransferase in several fungal species (see *Discussion*). The mutation results in an amino acid change at position 327 [alanine (GCC) to proline (CCC)] in the *A. niger* LaeA protein. The alanine residue at this position is conserved among 20 *Aspergillus* species (www.aspgd.broadinstitute.org).

### Complementation and disruption analysis

To show that the nonacidifying phenotype of the D15 mutant was caused by the mutation in *laeA*, the D15#26 mutant was complemented with the *laeA* gene. The *laeA* gene, including a ∼1000-nucleotide promoter and terminator region, was PCR-amplified and cloned into pAO4-13 containing the *pyrG* gene from *A. oryzae*. Transformation of the plasmid to MA273.1 or D15#26 restored the ability to acidify the medium, indicating that *laeA* complements the nonacidifying phenotype of the D15 mutant (Figure 2).

The *laeA* gene was also inactivated by targeted deletion. Bipartite gene deletion fragments were generated as described in *Materials and Methods* and transformed to AB4.1. Transformants were purified and analyzed for their acidification phenotype on MacConkey agar plates. Several transformants were isolated that did not acidify the medium, and these mutants were shown to be deleted in the *laeA* gene by Southern blot (Figure S1). The Δ*laeA* mutant was also cultivated in shake flask cultures as described for the D15 and the N402 strains (see above). Similar to the D15 mutant, the pH of the Δ*laeA* culture remained between 5.5 and 6.5 during the entire cultivation period. Organic acid analysis of the medium samples of the Δ*laeA* mutant also confirmed that the levels of citric and oxalic acid were reduced (data not shown). Both the complementation experiment and the targeted deletion of *laeA* show that the mutation in *laeA* in the D15 mutant is responsible for the acidification defect in the D15 mutant.

**Figure 3:**
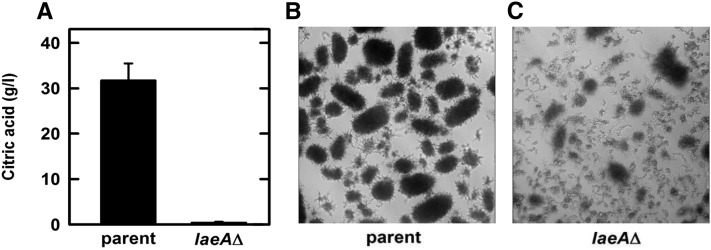
Citric acid levels of ATCC11414 and the ATCC11414Δ*laeA* strain. (A) Bar graph showing the results of citric acid production after 5 d in citric acid-production culture medium of the parent strain (ATCC11414*-kusA*), and the *laeA∆* mutant. The data for each strain are the average of at least three biological replicates. (B and C) The effects of *laeA* deletion on *A. niger* morphology. The conidia (1 × 10^6^ conidia/ml) were inoculated into 75 ml of citric acid-production medium in 250 ml silanized baffled flasks and shaken at 200 rpm at 30° for 5 d. Pellet formation from each culture was determined microscopically after 5 d of growth.

In order to assess the effect of *laeA* deletion under classical citric acid production conditions, we used strain ATCC11414, which is a spontaneous derivative of ATCC1015 (Dai *et al.* 2004; Baker 2006). Proper deletion of *laeA* in the ATCC11414 background was verified via diagnostic PCR (data not shown). Under low-manganese, high-glucose conditions, the parent strain can produce significant amounts of citrate. Deletion of *laeA* in this background resulted in a complete absence of citrate production in comparison to the parental strain, which made 30 g/l citric acid, indicating that LaeA is also required under high-citrate production conditions (Figure 3A). Deletion of *laeA* in the ATCC11414 background grown under citric acid-producing conditions altered the morphology of the culture. Whereas ATCC11414 formed pellets, which is the typical morphology during citric acid-producing conditions, pellets in the Δ*laeA* strain were smaller and the mycelium was much more dispersed (Figure 3B).

### Secondary metabolite profile of the laeA mutant in A. niger

**Figure 4:**
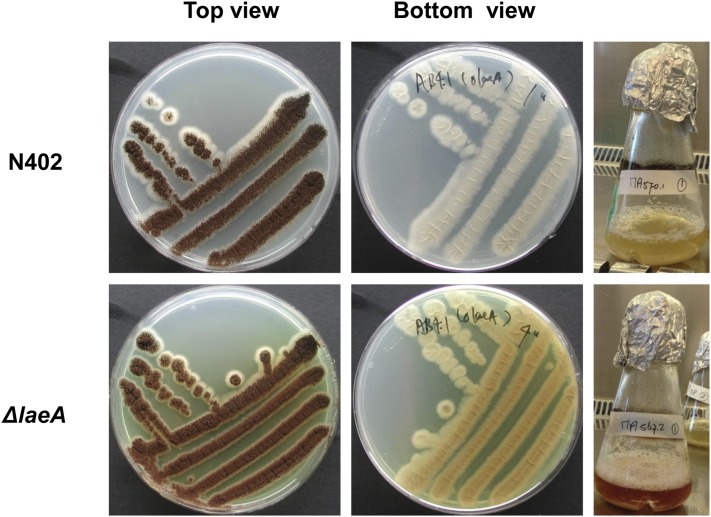
Secretion of secondary metabolites by the *A. niger laeA* mutant on minimal medium (MM) agar plates and MM-shake flask cultures. Spores of the wild-type and mutant were streaked to single colonies on MM agar plates and incubated at 30° for 5 d. For batch cultures, spores were inoculated at a density of 1 × 10^6^ spores/ml and grown at 30° for 5 d.

Previous studies have shown that the putative protein methyltransferase LaeA affects the expression of multiple secondary metabolite gene clusters in several fungi (Bok and Keller 2004; Sugui *et al.* 2007; Perrin *et al.* 2007; Bok *et al.* 2009; Butchko *et al.* 2012; Karimi-Aghcheh *et al.* 2013). We observed, when working with the D15 or Δ*laeA* strains, that plate-grown mycelium was yellowish and not greyish as seen in the wild-type, and that in submerged cultures of the Δ*laeA* mutant the medium turned purple (Figure 4). It is also apparent from Figure 4 that deletion of *laeA* did not result in an obvious growth defect under these conditions. To determine the role of *laeA* in *A. niger* in relation to secondary metabolite production, the production of secondary metabolites in wild-type and *laeA* mutants on three different media and culture conditions was analyzed. These conditions include submerged cultivation in nitrate-based minimal medium (subMM), and cultivation on solid media: Yeast Extract Sucrose (YES) agar and Czapek Yeast Autolysate (CYA) agar (see *Material and Methods*). We tested different media because it has been shown that these can have a pronounced effect on the production of secondary metabolites (Nielsen *et al.* 2011; Andersen *et al.* 2013). From this analysis, we could consistently identify seventeen compounds in the wild-type strains (Table 6). Nine of the seventeen compounds were detected under all three growth conditions, five compounds were detected on both YES and CYA agar, two compounds were detected on YES agar only, and one compound was only detected in subMM (Table 6). After establishing the secondary metabolite profile in the wild-type, it was possible to identify secondary metabolites whose production is affected by the absence of *laeA*, by comparing the profiles of the D15 strain and the Δ*laeA* strain (both *laeA*^-^) to the profiles of the original parental strain (N402) and the AB1.13 strain (*laeA^+^*). As indicated in Table 4, the presence or abundance of the majority of the secondary metabolites (11 out of 17) was not dramatically altered in the Δ*laeA* or D15 strain compared to the wild-type strains. Table S5 presents the identified compounds, including peak areas for each compound. Two compounds, BMS-192548 and aspernigrin A, were produced in much higher amounts in the *laeA* mutants compared to the wild-type strains, indicating that *laeA* has a repressive function for the expression of genes related to the production of these secondary metabolites. Three compounds, asperrubrol, atromentin and JBIR86, require LaeA, indicating that LaeA is involved in activating expression of the gene clusters responsible for the synthesis of these compounds. Interestingly, the requirement of LaeA for the production of these compounds is conditional and growth on YES agar medium bypasses the requirement of LaeA, as also observed by us for *A. fumigatus* (K. F. Nielsen and J. C. Frisvad, unpublished results). The production of tensidol B on CYA was absent in the Δ*laeA* mutant, while in the D15 mutant, which contains a point mutation in the *laeA* gene, tensidol B was still produced. The results indicate that the LaeA protein of *A. niger* can affect the expression of secondary metabolite gene clusters both positively and negatively.

**Table 6:** Identified secondary metabolites under different growth condition in *A. niger* and the effect of *laeA* inactivation on their production

Secondary metabolite	Remark
Aurasperone B	Production not affected by LaeA
Funalenone	Production not affected by LaeA
Kotanin*^a^*	Production of end product (Kotanin) not affected
Demethylkotanin*^a^*	Not present in Δ*laeA*
Orlandin*^a^*	Not present in Δ*laeA*
Asperrubrol	Production in subMM and CYA requires LaeA
Fumonisin B2/B4*^b^*	Production not affected by LaeA
Pyranopyrrol A	Production not affected by LaeA
Tensidol B	Production on CYA requires LaeA; production not affected in D15
Nigerazine	Production not affected by LaeA
Fungisporin A	Production not affected by LaeA
Expressed only on agar conditions	
Atromentin	Production on CYA requires LaeA
Pyranonigrin S	Production not affected by LaeA
Pestalamide C	Production not affected by LaeA
JBIR86	Production on CYA requires LaeA
Nigragilin	Production not affected by LaeA
Expressed only on YES	
Pyrophen	Production not affected by LaeA
Aspernigrin B	Production on CYA detected in Δ*laeA*
Expressed only in subMM	
BMS-192548*^c^*	Production 1000x increased in SubMM in Δ*laeA* and detected in CYA
Expressed only in Δ*laeA*	
Aspernigrin A	Production on CYA detected in Δ*laeA*

a Considered as one group of secondary metabolites.

b Fumonisin not detected in AB1.13, possibly because of mutation in the Fum gene cluster.

c Minor amount detected in N402 on YES agar, not detected in AB1.13.

## Discussion

Owing to its low production of proteases, the *A. niger* D15 mutant has been used in various studies of the production of heterologous proteins (Gordon *et al.* 2002; Rose and van Zyl 2002; Record *et al.* 2003; Benoit *et al.* 2007; Chimphango *et al.* 2012; Turbe-Doan *et al.* 2013; Benghazi *et al.* 2014; Piumi *et al.* 2014; Zwane *et al.* 2014). The D15 strain does not only contain a mutation in the protease regulator gene (*prtT*) (Punt *et al.* 2008) but also a mutation leading to a nonacidifying phenotype and, consequently, low levels of acid-induced proteases in the medium. Several attempts have been made to identify the mutation in the D15 mutant by complementation analysis, using a specific *A. niger* genomic cosmid library that has been successfully used before (Punt *et al.* 2008; Damveld *et al.* 2008; Meyer *et al.* 2009). However, complementation of the D15 nonacidifying phenotype was not successful, partly due to the problems involved in screening for complementation. We therefore decided to use whole genome sequencing to identify the responsible mutation. The strain lineage of the D15 mutant is rather complex (Figure 1), therefore, we used a bulk segregant approach, which narrows down the genomic region responsible for the phenotype. Whole genome sequencing in combination with genetic crosses to reduce the number of SNPs for further investigation has recently been used for mutant identification in *Neurospora crassa* (Pomraning *et al.* 2011), *Sordaria macrospora* (Nowrousian *et al.* 2012), and *A. nidulans* (Bok *et al.* 2014), either via a pooled-segregant approach (Pomraning *et al.* 2011; Nowrousian *et al.* 2012), or via successive backcrossings (Bok *et al.* 2014) using the sexual cycle. Since *A. niger* does not have a sexual cycle, which is normally used to obtain segregants, we employed the parasexual cycle of *A. niger* to generate segregants (Pontecorvo *et al.* 1953). For bulk segregant analysis, a pool of 78 nonacidifying segregants was used. The size of the pool turned out to be sufficient to narrow down the homozygous SNPs to a 1.6 Mb DNA region on chromosome II. This region contained only three fully homozygous SNPs (Table S4). Three other SNPs on chromosome II were clearly genetically coupled to the three fully conserved SNPs, but the coupling up to 97 to 98% indicated the occurrence of mitotic recombination in the diploid or during haploidization of the diploid. Since the occurrence of mitotic recombination is low, a mitotic cross-over involving the SNPs on chromosome II in a 1.4-MB region probably occurred only in a single segregant (out of 78). To further narrow down the number of relevant SNPs, a larger pool of segregants or the use of chemicals such as neomycin or 5-azacytidine (van de Vondervoort *et al.* 2007) to induce mitotic recombination might be used. However, in view of the relatively low number of SNPs found in the D15 mutant (52 in total), we were left with only a few candidate genes. It is interesting to note that the mutation at position 1762101 at chromosome II is located in gene An01g06900. This gene encodes a Zn(II)_2_Cys_6_ transcription factor (FumR), which is located in the fumonisin gene cluster. In the orthologous fumonisin gene cluster in *Fusarium verticillioides*, this transcription factor is required for fumonisin production (Brown *et al.* 2007). Secondary metabolite analysis of the AB1.13 and D15 revealed the absence of fumonisin in the AB1.13 and D15 mutants, and its presence in N402 and Δ*laeA* (Table S5). It is tempting to speculate that the mutation in the intron sequence of An01g06900 (already present in the AB1.13 mutant and its derivative D15) affects proper processing of mRNA, leading to a truncated and inactive FumR protein and an inability to produce fumonisin.

The role of LaeA in organic acid production, as shown in this paper, is not completely unprecedented. In *A. oryzae* it has been shown that deletion of the *laeA* homolog results in the loss of kojic acid production (Oda *et al.* 2011). The gene cluster likely to be involved in the synthesis of kojic acid production (AO09113000136, FDA-dependent oxidoreductase; AO09113000137, transcription factor; and AO09113000138, transporter protein) is severely down-regulated in the Δ*laeA* mutant of *A. oryzae*. A role for LaeA in citric acid production in *A. niger* is supported by the observation that overexpression of *A. nidulans laeA* in *A. niger* results in a 40% increase in citric acid production (Dai and Baker 2015). The increased production of citric acid upon *laeA* overexpression, and the reduced production in the *laeA* deletion strain, offer interesting possibilities to identify genes directly involved in citric acid production by transcriptomic or proteomic studies. Whether LaeA directly regulates genes involved in citric acid production, or whether its role is more indirect, *e.g.*, by affecting fungal morphology or by sensing the triggers that induce citric acid formation (low manganese, high glucose, etc.), is still not clear.

LaeA was initially identified as a regulator of secondary metabolism in *A. nidulans* (Bok and Keller 2004). Deletion of *laeA* in *A. nidulans* blocks the expression of several metabolic gene clusters, including gene clusters involved in sterigmatocystin, penicillin, and lovastatin biosynthesis, as grown on minimal media (Bok and Keller 2004). Its role as a global regulator of secondary metabolism has been established in various filamentous fungi, including *A. flavus* (Kale *et al.* 2008), *A. oryzae* (Oda *et al.* 2011), *A. fumigatus* (Perrin *et al.* 2007; Sugui *et al.* 2007), *Penicillium chrysogenum* (Kosalková *et al.* 2009), *P. citrinum* (Xing *et al.* 2010), *F. fujikuroi* (Wiemann *et al.* 2010), *F. verticillioides* (Butchko *et al.* 2012), *Trichoderma reesei* (Karimi-Aghcheh *et al.* 2013) and *Cochliobolus heterostrophus* (Wu *et al.* 2012). Yet another, but probably related function of LaeA, has to do with its role in *A. nidulans* as a member of the Velvet Complex, which consists of the LaeA, VeA, and VeB proteins, and controls asexual and sexual developmental pathways (Bayram and Braus 2012). Under light, LaeA is required for reduction of the VeA and VeB levels in order to stimulate asexual development. Conversely, in the absence of LaeA, VeA and VeB, protein levels are not repressed, leading to sexual development and the formation of cleistothecia (Bayram and Braus 2012). The role of LaeA in controlling gene expression is not necessarily restricted to secondary metabolites and development. Transcriptome analysis of the *laeA* mutant in *T. reesei* revealed that LaeA also controls the expression of extracellular enzymes (Seiboth *et al.* 2012), while LaeA in *P. chrysogenum* was found to affect chitinase expression (Kamerewerd *et al.* 2011). Whether and to what extent LaeA is involved in extracellular protein production in *A. niger* remains to be determined. As many of the extracellular enzymes in *A. niger* are highly expressed in an acidic environment, it is important to conduct these studies under pH-controlled conditions. It is further important to establish to what extent the differences in secondary metabolite production in *A. niger* are directly caused by *laeA* deletion, or whether the differences in secondary metabolite production are an indirect consequence of a different ambient pH.

Deletion of *laeA* in *A. niger* affects the production of several secondary metabolites. From the seventeen identified secondary metabolites, the production of six secondary metabolites was affected. Three compounds (aperrubrol, atromentin and JBIR86), from three very different pathways (aperrubrol is from the mixed polyketide-terpene pathway, atromentin from the shikimic acid pathway, whereas JBIR86 is amino acid-derived), were found to be produced in lower amounts in the *laeA* mutant, in agreement with the role of LaeA as a global regulator required for the biosynthesis of secondary metabolites (Bok and Keller, 2004; Bok *et al.* 2006, 2009). Interestingly, deletion of *laeA* also leads to increased production of two secondary metabolites (BMS-192548 and aspernigrin A). A similar role for LaeA as a negative regulator of the production of some secondary metabolites has also been reported for *C. heterostrophus* and *F. fujikuroi*, in which deletion of *laeA* results in increased melanin and bikaverin production, respectively, (Wu *et al.* 2012; Wiemann *et al.* 2010).

The link between LaeA and the production of citric acid or secondary metabolites changes our view of citric acid production in *A. niger* as a process belonging to primary metabolism. Both citrate and the oxalic acid precursor oxaloacetate play essential roles in the tricarboxylic acid cycle and are, therefore, genuine primary metabolites. However, our results point to a possible uncoupling of citric acid and oxalic acid production by alternative, LaeA-controlled metabolic pathways. Since growth of the Δ*laeA* mutant is not severely reduced, it is clear that primary metabolism in Δ*laeA* is not dramatically affected. LaeA’s specific role in citric acid production further suggests a need to consider the production of citric acid in *A. niger* as a process belonging to secondary metabolism. Oxalic acid production from oxaloacetate, without involvement of the tricarboxylic acid cycle, has also been previously reported (Kubicek *et al.* 1988). The chelating properties of both oxalic acid and citric acid and the corresponding ecological role of these acids in their natural habit, as well as the highly specific stress conditions that are required for citric acid production, support such a view. In addition, gene clusters are frequently involved in the production of secondary metabolites. Interestingly, gene clusters responsible for the production of itaconic acid and kojic acid have been found in *A. terreus* (Li *et al.* 2011) and *A. oryzae*, respectively (Oda *et al.* 2011), and this has supported the view that itaconic acid and kojic acids are secondary metabolites. With the *laeA* mutant and the *laeA*-overexpressing strain now available, we can search further for LaeA target genes involved in organic acid production in *A. niger* and other fungi.
